# Supplementary material for: Activity and Affinity of Pin1 Variants
Source: Molecules. 2019 Dec 20;25(1):36. doi: 10.3390/molecules25010036 (PMC6983177; doi:10.3390/molecules25010036)
Supplement: Supplementary file 1 [file molecules-25-00036-s001.pdf]

Supplemental Table S1: Pin1 Binding Affinity

Ligand	Pin1 Variant	K_D (uM)	Method	Conditions	Study
**Pintide (WFYpSPR)**	FL WT	200–400*	NMR titrations	100mM Imidazole, 100mM NaCl, 5mM DTT, 0.03% NaN_3, pH 6.6	Jacobs, D. M., Saxena, K., Vogtherr, M., Bernadó, P., Pons, M., & Fiebig, K. M. (2003). [1]
(WFYpSPFLE)	FL WT	17 ± 2.0*	Fluorescence Anisotropy	25mM HEPES-Na^+, 100mM NaCl, 1mM DTT, pH 7.5	Verdecia, M. A., Bowman, M. E., Lu, K. P., Hunter, T., and Noel, J. P. (2000). [2]
	WW	44 (9.5)	Fluorescence Anisotropy	25mM HEPES-Na^+, 100mM NaCl, 1mM DTT, pH 7.5	Verdecia, M. A., Bowman, M. E., Lu, K. P., Hunter, T., and Noel, J. P. (2000). [2]
	PPIase	86 (11)	Fluorescence Anisotropy	25mM HEPES-Na^+, 100mM NaCl, 1mM DTT, pH 7.5	Verdecia, M. A., Bowman, M. E., Lu, K. P., Hunter, T., and Noel, J. P. (2000). [2]
(WFYpSPRLKK)	PPIase	800 (150)	NMR titrations	5mM $NaPO_4$, 1mM TCEP, pH 6.7	Labeikovsky, W., Eisenmesser, E. Z., Bosco, D. A., & Kern, D. (2007). [3]
**CTD RNA Pol II (YpSPTpSPS)**	FL WT	200*	NMR titrations	100mM Imidazole, 100mM NaCl, 5mM DTT, 0.03% NaN_3, pH 6.6	Jacobs, D. M., Saxena, K., Vogtherr, M., Bernadó, P., Pons, M., & Fiebig, K. M. (2003). [1]
	FL WT	10 (0.83)	Fluorescence Anisotropy	25mM HEPES-Na^+, 100mM NaCl, 1mM DTT, pH 7.5	Verdecia, M. A., Bowman, M. E., Lu, K. P., Hunter, T., and Noel, J. P. (2000). [2]
	WW	34 (6.2)	Fluorescence Anisotropy	25mM HEPES-Na^+, 100mM NaCl, 1mM DTT, pH 7.5	Verdecia, M. A., Bowman, M. E., Lu, K. P., Hunter, T., and Noel, J. P. (2000). [2]
	FL R14A	WW: 5.8 (1.3)	Fluorescence Anisotropy	25mM HEPES-Na^+, 100mM NaCl, 1mM DTT, pH 7.5	Verdecia, M. A., Bowman, M. E., Lu, K. P., Hunter, T., and Noel, J. P. (2000). [2]
	FL S16H	WW: 174 (32)	Fluorescence Anisotropy	25mM HEPES-Na^+, 100mM NaCl, 1mM DTT, pH 7.5	Verdecia, M. A., Bowman, M. E., Lu, K. P., Hunter, T., and Noel, J. P. (2000). [2]
	FL S16A	WW: 28 (2.3)	Fluorescence Anisotropy	25mM HEPES-Na^+, 100mM NaCl, 1mM DTT, pH 7.5	Verdecia, M. A., Bowman, M. E., Lu, K. P., Hunter, T., and Noel, J. P. (2000). [2]
	FL R17A	WW: 63 (8)	Fluorescence Anisotropy	25mM HEPES-Na^+, 100mM NaCl, 1mM DTT, pH 7.5	Verdecia, M. A., Bowman, M. E., Lu, K. P., Hunter, T., and Noel, J. P. (2000). [2]

	FL Y23F	WW: 25 (1.7)	Fluorescence Anisotropy	25mM HEPES-Na^+, 100mM NaCl, 1mM DTT, pH 7.5	Verdecia, M. A., Bowman, M. E., Lu, K. P., Hunter, T., and Noel, J. P. (2000). [2]
	FL F25Y	WW: 12 (1.9)	Fluorescence Anisotropy	25mM HEPES-Na^+, 100mM NaCl, 1mM DTT, pH 7.5	Verdecia, M. A., Bowman, M. E., Lu, K. P., Hunter, T., and Noel, J. P. (2000). [2]
	FL F25L	WW: 2.7 (0.31)	Fluorescence Anisotropy	25mM HEPES-Na^+, 100mM NaCl, 1mM DTT, pH 7.5	Verdecia, M. A., Bowman, M. E., Lu, K. P., Hunter, T., and Noel, J. P. (2000). [2]
	FL F25V	WW: 3 (0.5)	Fluorescence Anisotropy	25mM HEPES-Na^+, 100mM NaCl, 1mM DTT, pH 7.5	Verdecia, M. A., Bowman, M. E., Lu, K. P., Hunter, T., and Noel, J. P. (2000). [2]
	FL F25A	WW: 7.6 (1.3)	Fluorescence Anisotropy	25mM HEPES-Na^+, 100mM NaCl, 1mM DTT, pH 7.5	Verdecia, M. A., Bowman, M. E., Lu, K. P., Hunter, T., and Noel, J. P. (2000). [2]
	FL W34F	WW: 63 (10)	Fluorescence Anisotropy	25mM HEPES-Na^+, 100mM NaCl, 1mM DTT, pH 7.5	Verdecia, M. A., Bowman, M. E., Lu, K. P., Hunter, T., and Noel, J. P. (2000). [2]
	FL W34A	WW: 180 (28)	Fluorescence Anisotropy	25mM HEPES-Na^+, 100mM NaCl, 1mM DTT, pH 7.5	Verdecia, M. A., Bowman, M. E., Lu, K. P., Hunter, T., and Noel, J. P. (2000). [2]
	PPIase	390 (82)	Fluorescence Anisotropy	25mM HEPES-Na^+, 100mM NaCl, 1mM DTT, pH 7.5	Verdecia, M. A., Bowman, M. E., Lu, K. P., Hunter, T., and Noel, J. P. (2000). [2]
**CTD RNA Pol II (YSPTpSPS)**	WW	35 (25)	ITC	20mM $NaPO_4$, pH 7	Jäger, M.; Zhang, Y.; Bieschke, J.; Nguyen, H.; Dendle, M.; … Kelly, J. W. (2006) [4]
	WW ^{17}-ADG^{20}	NB	ITC	20mM $NaPO_4$, pH 7	Jäger, M.; Zhang, Y.; Bieschke, J.; Nguyen, H.; Dendle, M.; … Kelly, J. W. (2006) [4]
	WW ^{17}-RDG^{20}	NB	ITC	20mM $NaPO_4$, pH 7	Jäger, M.; Zhang, Y.; Bieschke, J.; Nguyen, H.; Dendle, M.; … Kelly, J. W. (2006) [4]
	WW ^{17}-ARG^{20}	NB	ITC	20mM $NaPO_4$, pH 7	Jäger, M.; Zhang, Y.; Bieschke, J.; Nguyen, H.; Dendle, M.; … Kelly, J. W. (2006) [4]
	WW ^{17}--NG^{20}	NB	ITC	20mM $NaPO_4$, pH 7	Jäger, M.; Zhang, Y.; Bieschke, J.; Nguyen, H.; Dendle, M.; … Kelly, J. W. (2006) [4]
	WW ^{17}--RG^{20}	NB	ITC	20mM $NaPO_4$, pH 7	Jäger, M.; Zhang, Y.; Bieschke, J.; Nguyen, H.; Dendle, M.; … Kelly, J. W. (2006) [4]

**Cdc25c-T48 (VPRpTPV)**	FL WT	4.9 (1.1)	Fluorescence Anisotropy	25mM HEPES-Na^{+}, 100mM NaCl, 1mM DTT, pH 7.5	Verdecia, M. A., Bowman, M. E., Lu, K. P., Hunter, T., and Noel, J. P. (2000). [2]
	WW	7.7 (3.3)	Fluorescence Anisotropy	25mM HEPES-Na^{+}, 100mM NaCl, 1mM DTT, pH 7.5	Verdecia, M. A., Bowman, M. E., Lu, K. P., Hunter, T., and Noel, J. P. (2000). [2]
**pCDC25c (EQPLpTPVTDL)**	FL WT	117*	NMR titrations	100mM Imidazole, 100mM NaCl, 5mM DTT, 0.03% NaN_3, pH 6.6	Jacobs, D. M., Saxena, K., Vogtherr, M., Bernadó, P., Pons, M., & Fiebig, K. M. (2003). [1]
	FL WT	120*	NMR titrations	30mM Imidazole-D_4, 30mM NaCl, 0.03% NaN_3, 5mM DTT, pH 6.6	Namanja, A. T., Peng, T., Zintsmaster, J. S., Elson, A. C., Shakour, M. G., & Peng, J. W. (2007). [5]
	FL WT	8 (2)*	NMR titrations	30mM Imidazole-D_4, 30mM NaCl, 0.03% NaN_3, 5mM DTT, pH 6.6	Peng, J. W., Wilson, B. D., & Namanja, A. T. (2009). [6]
	FL WT	9 (1)*	NMR titrations	30mM Imidazole-D_4, 30mM NaCl, 0.03% NaN_3, 5mM DTT, pH 6.6	Wang, X., Mahoney, B. J., Zhang, M., Zintsmaster, J. S., & Peng, J. W. (2015). [7]
	FL WT	WW: 2.7 (0.7)	NMR titrations	30mM Imidazole-D_4, 30mM NaCl, 0.03% NaN_3, 5mM DTT, pH 6.6	Wilson, K. A., Bouchard, J. J., & Peng, J. W. (2013). [8]
	FL WT	WW: 6 (1)	NMR titrations	30mM Imidazole-D_4, 30mM NaCl, 0.03% NaN_3, 5mM DTT, pH 6.6	Peng, J. W. (2015). [9]
	FL WT	WW: 6.6 (2.2)	NMR titrations	50 mM Tris, 5 mM DTT, 0.3% NaN_3, pH 6.8	Zhu, W., Li, Y., Liu, M., Zhu, J., & Yang, Y. (2019). [10]
	WW	8.5 (7.7)	NMR titrations	10mM $NaPO_4$, 30mM NaCl, pH 7.0	Peng, T., Zintsmaster, J. S., Namanja, A. T., & Peng, J. W. (2007). [11]
	WW	13 (2)	ITC	30mM Imidazole-D_4, 30mM NaCl, 0.03% NaN_3, 5mM DTT,	Bouchard, J. (2014). [12]

				pH 6.6	
	WW	6.2 (2.3)	NMR titrations	50 mM Tris, 5 mM DTT, 0.3% NaN_3, pH 6.8	Zhu, W., Li, Y., Liu, M., Zhu, J., & Yang, Y. (2019). [10]
	WW	37.9 (2.4)	NMR titrations	30mM Imidazole-D_4, 30mM NaCl, 0.03% NaN_3, 5mM DTT, pH 6.6	Zhang, M., Case, D.A., Peng, J.W. (2018). [13]
	FL WT	PPIase: >120 (60)	NMR titrations	30mM Imidazole-D_4, 30mM NaCl, 0.03% NaN_3, 5mM DTT, pH 6.6	Peng, J. W. (2015). [9]
	FL WT	PPIase: 7.8 (0.4)	NMR titrations	30mM Imidazole-D_4, 30mM NaCl, 0.03% NaN_3, 5mM DTT, pH 6.6	Wilson, K. A., Bouchard, J. J., & Peng, J. W. (2013). [8]
	FL WT	PPIase ID interface:9.7 (2.0)	NMR titrations	30mM Imidazole-D_4, 30mM NaCl, 0.03% NaN_3, 5mM DTT, pH 6.6	Wilson, K. A., Bouchard, J. J., & Peng, J. W. (2013). [8]
	PPIase	10000	ITC	50mM Tris-HCl, 1mM DTT, 0.03% NaN_3, pH 6.8	Xu, N., Tochio, N., Wang, J., Tamari, Y., Uewaki, J. I., Utsunomiya-Tate, N., ... Tate, S. I. (2014). [14]
	PPIase	NB	NMR titrations	30mM Imidazole-D_4, 30mM NaCl, 0.03% NaN_3, 5mM DTT, pH 6.6	Peng, J. W. (2015). [9]
	PPIase	NB (>1mM)	ITC	30mM Imidazole-D_4, 30mM NaCl, 0.03% NaN_3, 5mM DTT, pH 6.6	Bouchard, J. (2014). [12]
	PPIase	1810 (290)	ITC	50mM Tris-HCl, 1mM DTT, 0.03% NaN_3, pH 6.8	Wang, J., Kawasaki, R., Uewaki, J. ichi, Rashid, A. U. R., Tochio, N., & Tate, S. ichi. (2017). [15]

	WW S19 deleted	47.7 (7.6)	NMR titrations	10mM $NaPO_4$, 30mM NaCl, pH 7.0	Peng, T., Zintsmaster, J. S., Namanja, A. T., & Peng, J. W. (2007). [11]
	FL I28A	55 (5)*	NMR titrations	30mM Imidazole-D_4, 30mM NaCl, 0.03% NaN_3, 5mM DTT, pH 6.6	Wang, X., Mahoney, B. J., Zhang, M., Zintsmaster, J. S., & Peng, J. W. (2015). [7]
	FL I28A	WW: 48.5 (4.9)	NMR titrations	30mM Imidazole-D_4, 30mM NaCl, 0.03% NaN_3, 5mM DTT, pH 6.6	Wilson, K. A., Bouchard, J. J., & Peng, J. W. (2013). [8]
	FL I28A	WW: 46 (5)	NMR titrations	30mM Imidazole-D_4, 30mM NaCl, 0.03% NaN_3, 5mM DTT, pH 6.6	Peng, J. W. (2015). [9]
	FL I28A	PPIase: 110 (10)	NMR titrations	30mM Imidazole-D_4, 30mM NaCl, 0.03% NaN_3, 5mM DTT, pH 6.6	Wilson, K. A., Bouchard, J. J., & Peng, J. W. (2013). [8]
	FL I28A	PPIase: >120 (60)	NMR titrations	30mM Imidazole-D_4, 30mM NaCl, 0.03% NaN_3, 5mM DTT, pH 6.6	Peng, J. W. (2015). [9]
	FL I28A	PPIase ID Interface: 65 (39)	NMR titrations	30mM Imidazole-D_4, 30mM NaCl, 0.03% NaN_3, 5mM DTT, pH 6.6	Wilson, K. A., Bouchard, J. J., & Peng, J. W. (2013). [8]
	WW Q33E	145.1 (2.8)	NMR titrations	30mM Imidazole-D_4, 30mM NaCl, 0.03% NaN_3, 5mM DTT, pH 6.6	Zhang, M., Case, D.A., Peng, J.W. (2018). [13]
	FL W34A	>2000*	NMR titrations	30mM Imidazole-D_4, 30mM NaCl, 0.03% NaN_3, 5mM DTT, pH 6.6	Wang, X., Mahoney, B. J., Zhang, M., Zintsmaster, J. S., & Peng, J. W. (2015). [7]
	FL dNSSSG (linker deletion)	WW: 7.3 (1.7)	NMR titrations	50 mM Tris, 5 mM DTT, 0.3% NaN_3, pH 6.8	Zhu, W., Li, Y., Liu, M., Zhu, J., & Yang, Y. (2019). [10]

	FL R68A/R69A	11 (1)*	NMR titrations	30mM Imidazole-D_4, 30mM NaCl, 0.03% NaN_3, 5mM DTT, pH 6.6	Wang, X., Mahoney, B. J., Zhang, M., Zintsmaster, J. S., & Peng, J. W. (2015). [7]
	PPIase C113D	NB	ITC	50mM Tris-HCl, 1mM DTT, 0.03% NaN_3, pH 6.8	Xu, N., Tochio, N., Wang, J., Tamari, Y., Uewaki, J. I., Utsunomiya-Tate, N., ... Tate, S. I. (2014). [14]
	PPIase S138A	1220 (880)	ITC	50mM Tris-HCl, 1mM DTT, 0.03% NaN_3, pH 6.8	Wang, J., Kawasaki, R., Uewaki, J. ichi, Rashid, A. U. R., Tochio, N., & Tate, S. ichi. (2017). [15]
**FFpSPR**	FL WT	203 (46)*	NMR titrations	30mM Imidazole-D_4, 30mM NaCl, 0.03% NaN_3, 5mM DTT, pH 6.6	Namanja, A. T., Wang, X. J., Xu, B., Mercedes-Camacho, A. Y., Wilson, B. D., Wilson, K. A., ... Peng, J. W. (2010). [16]
	FL WT	WW: 43 (14)	NMR titrations	30mM Imidazole-D_4, 30mM NaCl, 0.03% NaN_3, 5mM DTT, pH 6.6	Peng, J. W. (2015). [9]
	FL WT	WW: 184.3 (2.7)	NMR titrations	50 mM Tris, 5 mM DTT, 0.3% NaN_3, pH 6.8	Zhu, W., Li, Y., Liu, M., Zhu, J., & Yang, Y. (2019). [10]
	WW	246.8 (15.4)	NMR titrations	50 mM Tris, 5 mM DTT, 0.3% NaN_3, pH 6.8	Zhu, W., Li, Y., Liu, M., Zhu, J., & Yang, Y. (2019). [10]
	FL WT	PPIase: >80 (60)	NMR titrations	30mM Imidazole-D_4, 30mM NaCl, 0.03% NaN_3, 5mM DTT, pH 6.6	Peng, J. W. (2015). [9]
	PPIase	NB	NMR titrations	30mM Imidazole-D_4, 30mM NaCl, 0.03% NaN_3, 5mM DTT, pH 6.6	Peng, J. W. (2015). [9]
	FL dNSSSG (linker deletion)	WW: 139.1 (2)	NMR titrations	50 mM Tris, 5 mM DTT, 0.3% NaN_3, pH 6.8	Zhu, W., Li, Y., Liu, M., Zhu, J., & Yang, Y. (2019). [10]

**FFpSPR cis-locked**	FL WT	WW: NB	NMR titrations	30mM Imidazole-D_4, 30mM NaCl, 0.03% NaN_3, 5mM DTT, pH 6.6	Namanja, A. T., Wang, X. J., Xu, B., Mercedes-Camacho, A. Y., Wilson, K. A., Etzkorn, F. A., & Peng, J. W. (2011). [17]
	FL WT	WW: NB	NMR titrations	30mM Imidazole-D_4, 30mM NaCl, 0.03% NaN_3, 5mM DTT, pH 6.6	Peng, J. W. (2015). [9]
	FL WT	PPIase: 28	NMR titrations	30mM Imidazole-D_4, 30mM NaCl, 0.03% NaN_3, 5mM DTT, pH 6.6	Namanja, A. T., Wang, X. J., Xu, B., Mercedes-Camacho, A. Y., Wilson, K. A., Etzkorn, F. A., & Peng, J. W. (2011). [17]
	FL WT	PPIase: 28 (17)	NMR titrations	30mM Imidazole-D_4, 30mM NaCl, 0.03% NaN_3, 5mM DTT, pH 6.6	Peng, J. W. (2015). [9]
	PPIase	7	NMR titrations	30mM Imidazole-D_4, 30mM NaCl, 0.03% NaN_3, 5mM DTT, pH 6.6	Namanja, A. T., Wang, X. J., Xu, B., Mercedes-Camacho, A. Y., Wilson, K. A., Etzkorn, F. A., & Peng, J. W. (2011). [17]
	PPIase	7 (4)	NMR titrations	30mM Imidazole-D_4, 30mM NaCl, 0.03% NaN_3, 5mM DTT, pH 6.6	Peng, J. W. (2015). [9]
**FFpSPR *trans*-locked**	FL WT	78 (23)*	NMR titrations	30mM Imidazole-D_4, 30mM NaCl, 0.03% NaN_3, 5mM DTT, pH 6.6	Namanja, A. T., Wang, X. J., Xu, B., Mercedes-Camacho, A. Y., Wilson, B. D., Wilson, K. A., … Peng, J. W. (2010). [16]
	FL WT	WW: 53	NMR titrations	30mM Imidazole-D_4, 30mM NaCl, 0.03% NaN_3, 5mM DTT, pH 6.6	Namanja, A. T., Wang, X. J., Xu, B., Mercedes-Camacho, A. Y., Wilson, K. A., Etzkorn, F. A., & Peng, J. W. (2011). [17]
	FL WT	WW: 53 (16)	NMR titrations	30mM Imidazole-D_4, 30mM NaCl, 0.03% NaN_3, 5mM DTT, pH 6.6	Peng, J. W. (2015). [9]
	FL WT	PPIase: 37	NMR	30mM Imidazole-D_4, 30mM	Namanja, A. T., Wang, X. J., Xu, B., Mercedes-

			titrations	NaCl, 0.03% NaN_3, 5mM DTT, pH 6.6	Camacho, A. Y., Wilson, K. A., Etzkorn, F. A., & Peng, J. W. (2011). [17]
	FL WT	PPIase: 37 (13)	NMR titrations	30mM Imidazole-D_4, 30mM NaCl, 0.03% NaN_3, 5mM DTT, pH 6.6	Peng, J. W. (2015). [9]
	PPIase	66	NMR titrations	30mM Imidazole-D_4, 30mM NaCl, 0.03% NaN_3, 5mM DTT, pH 6.6	Namanja, A. T., Wang, X. J., Xu, B., Mercedes-Camacho, A. Y., Wilson, K. A., Etzkorn, F. A., & Peng, J. W. (2011). [17]
**Tau (SRSRpTPpSLPTPPTR)**	FL WT	160*	NMR titrations	50mM Tris, 100mM NaCl, pH 6.4	Smet, C., Wieruszeski, J.M., Buée, L., Landrieu. (2005). [18]
**suc-AEPF-pNA**	WT FL (open conformation)	1150 (370)*	NMR titrations	50mM KPO_4, pH 6.5	Matena, A., Sinnen, C., Van Den Boom, J., Wilms, C., Dybowski, J. N., Maltaner, R., … Bayer, P. (2013). [19]
	WT FL +2% PEG400 (closed conformation)	500 (150)*	NMR titrations	50mM KPO_4, pH 6.5	Matena, A., Sinnen, C., Van Den Boom, J., Wilms, C., Dybowski, J. N., Maltaner, R., … Bayer, P. (2013). [19]

* The domain for this NMR titration measurement was not specified, but is likely the binding of the WW domain.

NB: No binding was detected

Supplemental Table S2: Pin1 Activity using Exchange Spectroscopy (EXSY) at 295K

**Ligand**	**Pin1 Variant**	k_{ct} **(s^{-1})**	k_{tc} **(s^{-1})**	k_{EXSY} **(s^{-1})**	**Conditions**	**Study**
**pCDC25c (EQPLpSPVTDL)**	FL WT			8.8 (0.4)	30mM Imidazole-D_4, 30mM NaCl, 0.03% NaN_3, 5mM DTT, pH 6.6	Peng, J. W., Wilson, B. D., & Namanja, A. T. (2009). [6]

	FL WT			26.2 (1.0)	30mM Imidazole-D_4, 30mM NaCl, 0.03% NaN_3, 5mM DTT, pH 6.6	Mercedes-Camacho, A. Y., Mullins, A. B., Mason, M. D., Xu, G. G., Mahoney, B. J., Wang, X., … Etzkorn, F. A. (2013). [20]
	FL WT			31.3 (0.5)	30mM Imidazole-D_4, 30mM NaCl, 0.03% NaN_3, 5mM DTT, pH 6.6	Wilson, K. A., Bouchard, J. J., & Peng, J. W. (2013). [8]
	FL WT			33 (1)	30mM Imidazole-D_4, 30mM NaCl, 0.03% NaN_3, 5mM DTT, pH 6.6	Peng, J. W. (2015). [9]
	FL WT	31.3 (1) [#]	2 (0.06) [#]	33.3 (1.1)	30mM Imidazole-D_4, 30mM NaCl, 0.03% NaN_3, 5mM DTT, pH 6.6	Wang, X., Mahoney, B. J., Zhang, M., Zintsmaster, J. S., & Peng, J. W. (2015). [7]
	FL WT	40.5 (1.5)	4.1 (0.1)	44.6	50 mM Tris, 5 mM DTT, 0.3% NaN_3, pH 6.8	Zhu, W., Li, Y., Liu, M., Zhu, J., & Yang, Y. (2019). [10]
	FL WT	31.3 (1) [#]	2 (0.06) [#]	33.3 (1)	30mM Imidazole-D_4, 30mM NaCl, 0.03% NaN_3, 5mM DTT, pH 6.6	Mahoney, B. J., Zhang, M., Zintsmaster, J. S., & Peng, J. W. (2018). [21]
	PPIase			37 (1.1)	30mM Imidazole-D_4, 30mM NaCl, 0.03% NaN_3, 5mM DTT, pH 6.6	Peng, J. W., Wilson, B. D., & Namanja, A. T. (2009). [6]
	PPIase	51.6 (1.9)	6.6 (2.1)	58.2 (4.0)	50mM Tris-HCl, 1mM DTT, 0.03% NaN_3, pH 6.8	Xu, N., Tochio, N., Wang, J., Tamari, Y., Uewaki, J. I., Utsunomiya-Tate, N., … Tate, S. I. (2014). [14]
	PPIase			41 (1)	30mM Imidazole-D_4, 30mM NaCl, 0.03% NaN_3, 5mM DTT, pH 6.6	Peng, J. W. (2015). [9]
	PPIase	39.4 (1) [#]	2 (0.4) [#]	41 (0.04)	30mM Imidazole-D_4, 30mM NaCl, 0.03% NaN_3, 5mM DTT, pH 6.6	Wang, X., Mahoney, B. J., Zhang, M., Zintsmaster, J. S., & Peng, J. W. (2015). [7]
	PPIase	51.6 (1.9)	6.6 (2.1)	58.2 (4.0)	50mM Tris-HCl, 1mM DTT, 0.03% NaN_3, pH 6.8	Wang, J., Kawasaki, R., Uewaki, J. ichi, Rashid, A. U. R., Tochio, N., & Tate, S. ichi. (2017). [15]

	FL R17A/W34A	19.6 (0.5)	1.1 (0)	20.7	50 mM Tris, 5 mM DTT, 0.3% NaN_3, pH 6.8	Zhu, W., Li, Y., Liu, M., Zhu, J., & Yang, Y. (2019). [10]
	FL I28A			73 (2)	30mM Imidazole-D_4, 30mM NaCl, 0.03% NaN_3, 5mM DTT, pH 6.6	Wilson, K. A., Bouchard, J. J., & Peng, J. W. (2013). [8]
	FL I28A			73 (2)	30mM Imidazole-D_4, 30mM NaCl, 0.03% NaN_3, 5mM DTT, pH 6.6	Peng, J. W. (2015). [9]
	FL I28A	71.0 (1) #	2.43 (0.04) #	73 (2)	30mM Imidazole-D_4, 30mM NaCl, 0.03% NaN_3, 5mM DTT, pH 6.6	Wang, X., Mahoney, B. J., Zhang, M., Zintsmaster, J. S., & Peng, J. W. (2015). [7]
	FL W34A	17.2 (0.3) #	0.98 (0.01) #	18.2 (0.4)	30mM Imidazole-D_4, 30mM NaCl, 0.03% NaN_3, 5mM DTT, pH 6.6	Wang, X., Mahoney, B. J., Zhang, M., Zintsmaster, J. S., & Peng, J. W. (2015). [7]
	FL dNSSSG (linker deleted)	59.6 (1.8)	6.4 (0.2)	66	50 mM Tris, 5 mM DTT, 0.3% NaN_3, pH 6.8	Zhu, W., Li, Y., Liu, M., Zhu, J., & Yang, Y. (2019). [10]
	FL dSG (linker deleted)	43.5 (1.6)	4.6 (0.1)	48.1	50 mM Tris, 5 mM DTT, 0.3% NaN_3, pH 6.8	Zhu, W., Li, Y., Liu, M., Zhu, J., & Yang, Y. (2019). [10]
	FL dNSSSG (linker deleted)+W34A/R17A	73.3 (4.5)	1.5 (0.1)	74.8	50 mM Tris, 5 mM DTT, 0.3% NaN_3, pH 6.8	Zhu, W., Li, Y., Liu, M., Zhu, J., & Yang, Y. (2019). [10]
	FL R68A/R69A	ND	ND	ND	30mM Imidazole-D_4, 30mM NaCl, 0.03% NaN_3, 5mM DTT, pH 6.6	Wang, X., Mahoney, B. J., Zhang, M., Zintsmaster, J. S., & Peng, J. W. (2015). [7]
	FL S71E	11.2 (0.11) #	0.9 (0.01) #	12.1 (0.11)	30mM Imidazole-D_4, 30mM NaCl, 0.03% NaN_3, 5mM DTT, pH 6.6	Mahoney, B. J., Zhang, M., Zintsmaster, J. S., & Peng, J. W. (2018). [21]

	FL R74A	0.94 (1.83) #	0.05 (0.02) #	1 (1.83)	30mM Imidazole-D_4, 30mM NaCl, 0.03% NaN_3, 5mM DTT, pH 6.6	Mahoney, B. J., Zhang, M., Zintsmaster, J. S., & Peng, J. W. (2018). [21]
	FL R80Q	41.5 (6.1) #	1.02 (0.01) #	42.5 (6.1)	30mM Imidazole-D_4, 30mM NaCl, 0.03% NaN_3, 5mM DTT, pH 6.6	Mahoney, B. J., Zhang, M., Zintsmaster, J. S., & Peng, J. W. (2018). [21]
	FL D112N	3.81 (0.23) #	0.34 (0.01) #	4.14 (0.23)	30mM Imidazole-D_4, 30mM NaCl, 0.03% NaN_3, 5mM DTT, pH 6.6	Mahoney, B. J., Zhang, M., Zintsmaster, J. S., & Peng, J. W. (2018). [21]
	FL D112A	8.22 (0.07) #	0.59 (0.01) #	8.82 (0.07)	30mM Imidazole-D_4, 30mM NaCl, 0.03% NaN_3, 5mM DTT, pH 6.6	Mahoney, B. J., Zhang, M., Zintsmaster, J. S., & Peng, J. W. (2018). [21]
	PPIase C113A	1 (0.2)	0.1 (0.1)	1.1 (0.3)	50mM Tris-HCl, 1mM DTT, 0.03% NaN_3, pH 6.8	Wang, J., Kawasaki, R., Uewaki, J. ichi, Rashid, A. U. R., Tochio, N., & Tate, S. ichi. (2017). [15]
	PPIase C113D	0.7 (0.5)	0.1	0.8 (0.5)	50mM Tris-HCl, 1mM DTT, 0.03% NaN_3, pH 6.8	Xu, N., Tochio, N., Wang, J., Tamari, Y., Uewaki, J. I., Utsunomiya-Tate, N., ... Tate, S. I. (2014). [14]
	PPIase C113D	0.7 (0.5)	0.1	0.8 (0.5)	50mM Tris-HCl, 1mM DTT, 0.03% NaN_3, pH 6.8	Wang, J., Kawasaki, R., Uewaki, J. ichi, Rashid, A. U. R., Tochio, N., & Tate, S. ichi. (2017). [15]
	PPIase S138A	43.8 (0.7)	3.7 (2)	47.5 (2.7)	50mM Tris-HCl, 1mM DTT, 0.03% NaN_3, pH 6.8	Wang, J., Kawasaki, R., Uewaki, J. ichi, Rashid, A. U. R., Tochio, N., & Tate, S. ichi. (2017). [15]
**FFpSPR**	FL WT	87 (4)			30mM Imidazole-D_4, 30mM NaCl, 0.03% NaN_3, 5mM DTT, pH 6.6	Namanja, A. T., Wang, X. J., Xu, B., Mercedes-Camacho, A. Y., Wilson, B. D., Wilson, K. A., ... Peng, J. W. (2010). [16]
	FL WT			44 (3)	30mM Imidazole-D_4, 30mM NaCl, 0.03% NaN_3, 5mM DTT, pH 6.6	Peng, J. W. (2015). [9]
	FL WT	93.5 (3.7)	4.1 (0.2)	97.6	50 mM Tris, 5 mM DTT, 0.3% NaN_3, pH 6.8	Zhu, W., Li, Y., Liu, M., Zhu, J., & Yang, Y. (2019). [10]
	PPIase			91 (9)	30mM Imidazole-D_4, 30mM	Peng, J. W. (2015). [9]

					NaCl, 0.03% NaN_3, 5mM DTT, pH 6.6	
	FL W34A	40.7 (1)	4.3 (0.1)	45	50 mM Tris, 5 mM DTT, 0.3% NaN_3, pH 6.8	Zhu, W., Li, Y., Liu, M., Zhu, J., & Yang, Y. (2019). [10]
**Tau (SRSRpTPpSLPTPPTR)**	FL WT	0.8			25mM Tris, 50mM NaCl, pH 6.4	Smet, C., Wieruszeski, J.M., Buée, L., Landrieu. (2005). [18]
	PPIase	0.1			25mM Tris, 50mM NaCl, pH 6.4	Smet, C., Wieruszeski, J.M., Buée, L., Landrieu. (2005). [18]

ND: No isomerization was detected.

[#] Values in original papers were switched and have since been corrected

Supplemental Table S3: Pin1 Activity using Trypsin/Chymotrypsin Degradation Assay

**Ligand**	**Pin1 Variant**	**K_m (uM)**	**k_{cat}/K_m ($mM^{-1}s^{-1}$)**	**Conditions**	**Study**
**suc-AEPF-pNA**	WT FL	172		30mM Imidazole, 30mM NaCl, 0.03% NaN_3, 5mM DTT, pH 6.6	Namanja, A. T., Peng, T., Zintsmaster, J. S., Elson, A. C., Shakour, M. G., & Peng, J. W. (2007). [5]
	WT FL	310 (72)	440 (150)	50mM HEPES, 100mM NaCl, 5mM NaN_3, pH 7.5	Behrsin, C. D., Bailey, M. L., Bateman, K. S., Hamilton, K. S., Wahl, L. M., Brandl, C. J., … Litchfield, D. W. (2007). [22]
	WT FL		4250 (213)	30mM Imidazole-D_4, 30mM NaCl, 0.03% NaN_3, 5mM DTT, pH 6.6	Wilson, K. A., Bouchard, J. J., & Peng, J. W. (2013). [8]
	PPIase		380 (20)	5mM $NaPO_4$, 1mM DTT, pH 6.7	Labeikovsky, W., Eisenmesser, E. Z., Bosco, D. A., & Kern, D. (2007). [3]

	I28A		2724 (140)	30mM Imidazole-D_4, 30mM NaCl, 0.03% NaN_3, 5mM DTT, pH 6.6	Wilson, K. A., Bouchard, J. J., & Peng, J. W. (2013). [8]
	K63A	700 (190)	16 (7.2)	50mM HEPES, 100mM NaCl, 5mM NaN_3, pH 7.5	Behrsin, C. D., Bailey, M. L., Bateman, K. S., Hamilton, K. S., Wahl, L. M., Brandl, C. J., ... Litchfield, D. W. (2007). [22]
	R68/R69A	170 (54)	110 (47)	50mM HEPES, 100mM NaCl, 5mM NaN_3, pH 7.5	Behrsin, C. D., Bailey, M. L., Bateman, K. S., Hamilton, K. S., Wahl, L. M., Brandl, C. J., ... Litchfield, D. W. (2007). [22]
	C113D	370 (130)	78 (42)	50mM HEPES, 100mM NaCl, 5mM NaN_3, pH 7.5	Behrsin, C. D., Bailey, M. L., Bateman, K. S., Hamilton, K. S., Wahl, L. M., Brandl, C. J., ... Litchfield, D. W. (2007). [22]
	C113S	410 (93)	9 (3.2)	50mM HEPES, 100mM NaCl, 5mM NaN_3, pH 7.5	Behrsin, C. D., Bailey, M. L., Bateman, K. S., Hamilton, K. S., Wahl, L. M., Brandl, C. J., ... Litchfield, D. W. (2007). [22]
**WFYpSPR-pNA**	WT FL		4900 (450)	50mM HEPES, 100mM NaCl, 5mM NaN_3, pH 7.5	Behrsin, C. D., Bailey, M. L., Bateman, K. S., Hamilton, K. S., Wahl, L. M., Brandl, C. J., ... Litchfield, D. W. (2007). [22]
	C113D		1400 (160)	50mM HEPES, 100mM NaCl, 5mM NaN_3, pH 7.5	Behrsin, C. D., Bailey, M. L., Bateman, K. S., Hamilton, K. S., Wahl, L. M., Brandl, C. J., ... Litchfield, D. W. (2007). [22]
	C113S		58 (2.0)	50mM HEPES, 100mM NaCl, 5mM NaN_3, pH 7.5	Behrsin, C. D., Bailey, M. L., Bateman, K. S., Hamilton, K. S., Wahl, L. M., Brandl, C. J., ... Litchfield, D. W. (2007). [22]
	WT FL		20160	Not included in publication	Yaffe, M. B.; Schutkowski, M.; Shen, M.; Zhou, X. Z.; Stukenberg, P. T.; Rahfeld, J. U.; Xu, J.; Kuang, J.; Kirschner, M. W.; Fischer, G.; Cantley, L. C.; Lu, K. P. (1997). [23]
	WT FL		3200 (160)*	50mM HEPES, 100mM NaCl, 5mM NaN_3, pH 7.5	Bailey, M. L.; Shilton, B. H.; Brandl, C. J.; Litchfield, D. W. (2008).[24]
	FL H157S		2624 (131)*	50mM HEPES, 100mM NaCl, 5mM NaN_3, pH 7.5	Bailey, M. L.; Shilton, B. H.; Brandl, C. J.; Litchfield, D. W. (2008).[24]
	FL H157A		1952 (98)*	50mM HEPES, 100mM NaCl, 5mM NaN_3, pH 7.5	Bailey, M. L.; Shilton, B. H.; Brandl, C. J.; Litchfield, D. W. (2008).[24]
	FL 59S		1600 (80)*	50mM HEPES, 100mM NaCl, 5mM NaN_3, pH 7.5	Bailey, M. L.; Shilton, B. H.; Brandl, C. J.; Litchfield, D. W. (2008).[24]

	FL H59L/H157A		1318 (66)*	50mM HEPES, 100mM NaCl, 5mM NaN_3, pH 7.5	Bailey, M. L.; Shilton, B. H.; Brandl, C. J.; Litchfield, D. W. (2008).[24]
	FL H59A		1152 (58)*	50mM HEPES, 100mM NaCl, 5mM NaN_3, pH 7.5	Bailey, M. L.; Shilton, B. H.; Brandl, C. J.; Litchfield, D. W. (2008).[24]
	FL H59N		960 (48)*	50mM HEPES, 100mM NaCl, 5mM NaN_3, pH 7.5	Bailey, M. L.; Shilton, B. H.; Brandl, C. J.; Litchfield, D. W. (2008).[24]
	FL H157L		736 (37)*	50mM HEPES, 100mM NaCl, 5mM NaN_3, pH 7.5	Bailey, M. L.; Shilton, B. H.; Brandl, C. J.; Litchfield, D. W. (2008).[24]
	FL H59L/H157L		480 (24)*	50mM HEPES, 100mM NaCl, 5mM NaN_3, pH 7.5	Bailey, M. L.; Shilton, B. H.; Brandl, C. J.; Litchfield, D. W. (2008).[24]
	FL H157N		448 (22)*	50mM HEPES, 100mM NaCl, 5mM NaN_3, pH 7.5	Bailey, M. L.; Shilton, B. H.; Brandl, C. J.; Litchfield, D. W. (2008).[24]
	FL H157F		192 (10)*	50mM HEPES, 100mM NaCl, 5mM NaN_3, pH 7.5	Bailey, M. L.; Shilton, B. H.; Brandl, C. J.; Litchfield, D. W. (2008).[24]
	FL H59L/H157F		160 (8)*	50mM HEPES, 100mM NaCl, 5mM NaN_3, pH 7.5	Bailey, M. L.; Shilton, B. H.; Brandl, C. J.; Litchfield, D. W. (2008).[24]
	FL H59L/H157S		160 (8)*	50mM HEPES, 100mM NaCl, 5mM NaN_3, pH 7.5	Bailey, M. L.; Shilton, B. H.; Brandl, C. J.; Litchfield, D. W. (2008).[24]
	FL H59L		96 (5)*	50mM HEPES, 100mM NaCl, 5mM NaN_3, pH 7.5	Bailey, M. L.; Shilton, B. H.; Brandl, C. J.; Litchfield, D. W. (2008).[24]
	FL C113S		26 (1)*	50mM HEPES, 100mM NaCl, 5mM NaN_3, pH 7.5	Bailey, M. L.; Shilton, B. H.; Brandl, C. J.; Litchfield, D. W. (2008).[24]
	FL H59L/H157N		15 (1)*	50mM HEPES, 100mM NaCl, 5mM NaN_3, pH 7.5	Bailey, M. L.; Shilton, B. H.; Brandl, C. J.; Litchfield, D. W. (2008).[24]
WFYSPR-pNA	WT FL		170	Not included in publication	Yaffe, M. B.; Schutkowski, M.; Shen, M.; Zhou, X. Z.;

(unphosporylated)					Stukenberg, P. T.; Rahfeld, J. U.; Xu, J.; Kuang, J.; Kirschner, M. W.; Fischer, G.; Cantley, L. C.; Lu, K. P. (1997). [23]

*These measurement values were not explicit in the published paper, as they were only reported in a bar graph. These values reported here are inferences based off the value in the bar graph, with an error of ± 5%.

References

1. Jacobs, D. M.; Saxena, K.; Vogtherr, M.; Bernadó, P.; Pons, M.; Fiebig, K. M. Peptide binding induces large scale changes in inter-domain mobility in human Pin1. *J. Biol. Chem.* **2003**, *278*, 26174–26182, doi:10.1074/jbc.M300796200.
2. Verdecia, M. A.; Bowman, M. E.; Lu, K. P.; Hunter, T.; Noel, J. P. Structural basis for phosphoserine-proline recognition by group IV WW domains. *Nat. Struct. Biol.* **2000**, *7*, 639–643, doi:10.1038/77929.
3. Labeikovsky, W.; Eisenmesser, E. Z.; Bosco, D. A.; Kern, D. Structure and Dynamics of Pin1 During Catalysis by NMR. *J. Mol. Biol.* **2007**, *367*, 1370–1381, doi:10.1016/j.jmb.2007.01.049.
4. Jäger, M.; Zhang, Y.; Bieschke, J.; Nguyen, H.; Dendle, M.; Bowman, M. E.; Noel, J. P.; Gruebele, M.; Kelly, J. W. Structure-function-folding relationship in a WW domain. *Proc. Natl. Acad. Sci. U. S. A.* **2006**, *103*, 10648–53, doi:10.1073/pnas.0600511103.
5. Namanja, A. T.; Peng, T.; Zintsmaster, J. S.; Elson, A. C.; Shakour, M. G.; Peng, J. W. Substrate Recognition Reduces Side-Chain Flexibility for Conserved Hydrophobic Residues in Human Pin1. *Structure* **2007**, *15*, 313–327, doi:10.1016/j.str.2007.01.014.
6. Peng, J. W.; Wilson, B. D.; Namanja, A. T. Mapping the dynamics of ligand reorganization via 13CH3 and 13CH2 relaxation dispersion at natural abundance. *J. Biomol. NMR* **2009**, *45*, 171–83, doi:10.1007/s10858-009-9349-4.
7. Wang, X.; Mahoney, B. J.; Zhang, M.; Zintsmaster, J. S.; Peng, J. W. Negative Regulation of Peptidyl-Prolyl Isomerase Activity by Interdomain Contact in Human Pin1. *Structure* **2015**, *23*, 2224–2233, doi:10.1016/j.str.2015.08.019.
8. Wilson, K. A.; Bouchard, J. J.; Peng, J. W. Interdomain interactions support interdomain communication in human pin1. *Biochemistry* **2013**, *52*, 6968–6981, doi:10.1021/bi401057x.
9. Peng, J. W. Investigating dynamic interdomain allostery in Pin1. *Biophys. Rev.* **2015**, *7*, 239–249, doi:10.1007/s12551-015-0171-9.
10. Zhu, W.; Li, Y.; Liu, M.; Zhu, J.; Yang, Y. Uncorrelated Effect of Interdomain Contact on Pin1 Isomerase Activity Reveals Positive Catalytic Cooperativity. *J. Phys. Chem. Lett.* **2019**, *10*, 1272–1278, doi:10.1021/acs.jpclett.9b00052.
11. Peng, T.; Zintsmaster, J. S.; Namanja, A. T.; Peng, J. W. Sequence-specific dynamics modulate recognition specificity in WW domains. *Nat. Struct. Mol. Biol.* **2007**, *14*, 325–331, doi:10.1038/nsmb1207.
12. Bouchard, J. J. Ensemble Interpretation of Domain Mobility in Modular Protein Pin1 by NMR and Molecular Dynamics, University of Notre Dame, 2014.
13. Zhang, M.; Case, D. A.; Peng, J. W. Propagated Perturbations from a Peripheral Mutation Show Interactions Supporting WW Domain Thermostability. *Structure* **2018**, *26*, 1474-1485.e5, doi:10.1016/j.str.2018.07.014.
14. Xu, N.; Tochio, N.; Wang, J.; Tamari, Y.; Uewaki, J. I.; Utsunomiya-Tate, N.; Igarashi, K.; Shiraki, T.; Kobayashi, N.; Tate, S. I. The C113D mutation in human Pin1 causes allosteric structural changes in the phosphate binding pocket of the ppiase domain through the tug of

war in the dual-histidine motif. *Biochemistry* **2014**, *53*, 5568–5578, doi:10.1021/bi5007817.

15. Wang, J.; Kawasaki, R.; Uewaki, J. ichi; Rashid, A. U. R.; Tochio, N.; Tate, S. ichi Dynamic allostery modulates catalytic activity by modifying the hydrogen bonding network in the catalytic site of human Pin1. *Molecules* **2017**, *22*, doi:10.3390/molecules22060992.
16. Namanja, A. T.; Wang, X. J.; Xu, B.; Mercedes-Camacho, A. Y.; Wilson, B. D.; Wilson, K. A.; Etzkorn, F. A.; Peng, J. W. Toward flexibility-activity relationships by NMR spectroscopy: Dynamics of Pin1 ligands. *J. Am. Chem. Soc.* **2010**, *132*, 5607–5609, doi:10.1021/ja9096779.
17. Namanja, A. T.; Wang, X. J.; Xu, B.; Mercedes-Camacho, A. Y.; Wilson, K. A.; Etzkorn, F. A.; Peng, J. W. Stereospecific gating of functional motions in Pin1. *Proc. Natl. Acad. Sci.* **2011**, *108*, 12289–12294, doi:10.1073/pnas.1019382108.
18. Smet, C.; Wieruszeski, J.-M.; Buée, L.; Landrieu, I.; Lippens, G. Regulation of Pin1 peptidyl-prolyl *cis/trans* isomerase activity by its WW binding module on a multi-phosphorylated peptide of Tau protein. *FEBS Lett.* **2005**, *579*, 4159–4164, doi:10.1016/j.febslet.2005.06.048.
19. Matena, A.; Sinnen, C.; Van Den Boom, J.; Wilms, C.; Dybowski, J. N.; Maltaner, R.; Mueller, J. W.; Link, N. M.; Hoffmann, D.; Bayer, P. Transient domain interactions enhance the affinity of the mitotic regulator pin1 toward phosphorylated peptide ligands. *Structure* **2013**, *21*, 1769–1777, doi:10.1016/j.str.2013.07.016.
20. Mercedes-Camacho, A. Y.; Mullins, A. B.; Mason, M. D.; Xu, G. G.; Mahoney, B. J.; Wang, X.; Peng, J. W.; Etzkorn, F. A. Kinetic isotope effects support the twisted amide mechanism of Pin1 peptidyl-prolyl isomerase. *Biochemistry* **2013**, *52*, 7707–7713, doi:10.1021/bi400700b.
21. Mahoney, B. J.; Zhang, M.; Zintsmaster, J. S.; Peng, J. W. Extended Impact of Pin1 Catalytic Loop Phosphorylation Revealed by S71E Phosphomimetic. *J. Mol. Biol.* **2018**, *430*, 710–721, doi:10.1016/J.JMB.2017.12.021.
22. Behrsin, C. D.; Bailey, M. L.; Bateman, K. S.; Hamilton, K. S.; Wahl, L. M.; Brandl, C. J.; Shilton, B. H.; Litchfield, D. W. Functionally Important Residues in the Peptidyl-prolyl Isomerase Pin1 Revealed by Unigenic Evolution. *J. Mol. Biol.* **2007**, *365*, 1143–1162, doi:10.1016/j.jmb.2006.10.078.
23. Yaffe, M. B.; Schutkowski, M.; Shen, M.; Zhou, X. Z.; Stukenberg, P. T.; Rahfeld, J. U.; Xu, J.; Kuang, J.; Kirschner, M. W.; Fischer, G.; Cantley, L. C.; Lu, K. P. Sequence-specific and phosphorylation dependent proline isomerization: A potential mitotic regulatory mechanism. *Science (80-.).* **1997**, *278*, 1957–1960, doi:10.1126/science.278.5345.1957.
24. Bailey, M. L.; Shilton, B. H.; Brandl, C. J.; Litchfield, D. W. The dual histidine motif in the active site of Pin1 has a structural rather than catalytic role. *Biochemistry* **2008**, *47*, 11481–11489, doi:10.1021/bi800964q.
